# Early Psychological Intervention After Rape: A Feasibility Study

**DOI:** 10.3389/fpsyg.2020.01595

**Published:** 2020-07-08

**Authors:** Maria Bragesjö, Karin Larsson, Lisa Nordlund, Therese Anderbro, Erik Andersson, Anna Möller

**Affiliations:** ^1^Department of Clinical Neuroscience, Division of Psychology, Karolinska Institutet, Stockholm, Sweden; ^2^Department of Obstetrics and Gynecology, Emergency Clinic for Rape Victims, Stockholm South Hospital, Stockholm, Sweden; ^3^Department of Psychology, Stockholm University, Stockholm, Sweden; ^4^Department of Clinical Sciences, Danderyd Hospital, Karolinska Institutet, Stockholm, Sweden; ^5^Department of Clinical Science and Education, Stockholm South Hospital, Karolinska Institutet, Stockholm, Sweden

**Keywords:** prolonged exposure, prevention, post-traumatic stress disorder, early intervention, acute stress disorder, rape, sexual assault

## Abstract

Rape is the most common trauma leading to post-traumatic stress disorder (PTSD) among women, with a conditioned prevalence of up to 50%. PTSD is considered to be a lethal condition associated with increased risk of suicide, drug- and alcohol dependence, neurological- and vascular problems, as well as sick leave. Given the scope of this problem, novel and swiftly delivered interventions for this large vulnerable population are clearly warranted. One previous trial conducted in the United States (*N* = 137) showed that an adapted brief version of prolonged exposure (PE) to the fearful memory of the event and situations, provided in the immediate aftermath after trauma (<72 h after a traumatic event), was effective in reducing early PTSD symptoms in rape victims. The aims of the present study were to adapt the brief PE protocol to a Swedish context and investigate its feasibility and delivery in 10 executive patients recruited at the Emergency Clinic for Rape Victims in Stockholm. Ten participants were provided with three sessions of early PE with overall successful results in terms of session attendance, home-work compliance, and also symptom reduction of PTSD and depressive symptoms. However, only a fraction of the screened patients at the Emergency Clinic (5.2%) were eligible to be included in the study, where the majority (40%) were excluded due to the time criteria of 72 h. In this article, we will present detailed results of the intervention and elaborate on how to increase feasibility of preventive interventions for rape victims. In the current form, providing PE with the strict time criteria was not feasible in the clinical setting that constitutes the Emergency Department for rape.

## Introduction

Sexual assault, including sexual abuse, rape, intimate partner, and sexual violence, is a global public health concern affecting an estimated 12% of women around the world (Scott et al., 2018). In a recent population-based study from Sweden, 20% of women reported ever having been sexually assaulted (NCK, 2014). Sexual assault can lead to a variety of problems including lifetime diagnosis of anxiety disorders, depression, post-traumatic stress disorder (PTSD), eating- and sleep disorders, suicide attempts (Chen et al., 2010; Dartnall and Jewkes, 2013), gynecological problems, neurological, vascular, respiratory, gastrointestinal, and autoimmune diseases (McFarlane et al., 1994; Jina and Thomas, 2013). Sexual assault may also lead to increased alcohol- and marijuana misuse (Dartnall and Jewkes, 2013), which in turn can increase the risk of additional sexual revictimization (Hannan et al., 2017). Importantly, a recent large-scale epidemiological study from Iceland showed an increased risk for negative effects on both maternal health as well as fetal development among women with a history of sexual assault (Gisladottir et al., 2014). This is an important finding given that as much as one out of 12 pregnant women has experienced a sexual assault during her lifetime (Stenson et al., 2003).

Among women who have been exposed to rape, as much as 19–50% develop PTSD (Breslau et al., 1998; Kessler et al., 2005; Tiihonen et al., 2014; Liu et al., 2017). Rape may also cause severe social consequences due to stigma associated with the event, and <16% of cases are actually reported to the police (Wolitzky-Taylor et al., 2011). Given the scope of this problem, it is imperative to develop psychological interventions in the early aftermath of rape to reduce early symptoms which may also prevent the development of mental health problems.

There have been some attempts to provide rape victims with early trauma-focused psychological interventions. Resnick et al. (1999) attempted to reduce trauma reactions in rape victims by providing a 17-min video-based intervention provided prior to the forensic rape examination. The intervention consisted of information about the forensic examination, psychoeducation about common trauma-related reactions (intrusions of the event, marijuana use, depressive symptoms), and coping strategies to tackle these symptoms. One pilot trial (*N* = 124) indicated that this brief video intervention could significantly reduce both marijuana misuse at 6 weeks follow-up (Acierno et al., 2003) and distress before the forensic examination (Resnick H. et al., 2007). A subsequent trial in 2007 (*N* = 140) by the same research group found that women with a pre-assault history of high marijuana use who were randomized to the video intervention had lower marijuana misuse scores from baseline to the 6-months follow-up (Resnick H. S. et al., 2007). An even briefer version of the video-intervention (9 min) was tested in another trial in 2015 (*N* = 164) where women randomized to the video condition had fewer anxiety symptoms at the 2-month follow-up but no main effects were found on PTSD symptoms (Miller et al., 2015). More recently, the same research group used the brief intervention (*N* = 154) compared to treatment as usual and an active control condition (pleasant imagery and relaxation instruction). Results showed that the video intervention had some efficacy in reducing substance use in rape victims with a history of sexual assault (Walsh et al., 2017).

There has also been one attempt to scale up the dosage of early psychological interventions for rape victims. Rothbaum et al. (2012) randomized 137 patients seeking medical care at an emergency hospital within the first 72 h of trauma to either a brief version of prolonged exposure (PE; the first-line treatment for PTSD) or to assessment only. The PE intervention consisted of three 60-min sessions scheduled during a 2-week period. The main intervention was based on repeated exposure to the fearful memory of the event (imaginal exposure) and situations, places, persons, and activities that has become associated with fear since the event (*in vivo* exposure). Results showed that PE was especially effective in reducing symptoms of post-traumatic stress among the subsample of rape victims (*n* = 47, 28% of the original sample) with a medium effect size (*d* = 0.7) at 12 weeks. A more recent study did not replicate these promising findings when comparing the three session protocol against one session and assessment only (Maples-Keller et al., 2020). However, this study had a much lower proportion of rape victims, and there was no separate subgroup analysis for this particular trauma type.

To summarize, rape is common in the population, and it is of great importance to develop effective early interventions that can be provided to swiftly reach rape victims and thus reduce the development of long-term mental health problems such as PTSD. There have been some attempts of providing early interventions to women experiencing rape where a brief version of PE has shown promising results (Rothbaum et al., 2012). The aim of this study was to translate and test the feasibility of the brief PE protocol at the Emergency Clinic for Rape Victims in Stockholm, Sweden. We hypothesized that PE, provided in the early aftermath of rape, would be an acceptable and deliverable early treatment for rape victims at the clinic.

## Materials and Methods

### Trial Design

The study used an open trial design. Ten consecutive patients attending the Emergency Clinic for Rape Victims at Stockholm South Hospital, Sweden, were included during the study period (170111-170404). The clinic is one of the largest sexual assault centers in Europe with an intake of about 800 patients each year. Medical care, forensic examination, and psychological help are offered to patients in Stockholm county over 13 years who seek help within 1 month after the rape. Neither medical nor psychological help render any fee for the patient but is solely financed by the Stockholm county. The study was approved by the Regional Ethical Review Board in Stockholm, Sweden (ID:2016/2194–31).

### Participants

Eligible participants were patients of at least 18 years of age attending at the Emergency Clinic for Rape Victims at Stockholm South Hospital, Sweden, within 72 h (which was the same time criteria as in the Rothbaum et al. trial) after experiencing a traumatic event meeting the DSM-5 criterion A for PTSD (exposed to actual or threatened death, serious injury, or sexual violence) and who had memories from the event. Exclusion criteria were (i) ongoing suicidal ideation or attempted suicide within the last 2 months, (ii) ongoing self-harm behavior, (iii) ongoing intoxication, (iv) other serious psychiatric comorbidity (ongoing psychotic symptoms or manic episode), (v) low cognitive capacity, (vi) not fluent in Swedish, and (vii) subjected to ongoing violence or threat.

### Procedure

Recruitment was conducted in two steps. Each morning, a clinical psychologist or licensed psychotherapist did a pre-selection screening in the medical records of newly arrived patients. Eligible patients were asked to participate in the study at a routine appointment with either a clinical psychologist or a licensed psychotherapist. Patients who did not come to the routine appointment were instead reached by telephone. Patients signed informed consent at the assessment appointment and also completed the baseline battery consisting of Immediate Stress Reaction Checklist (ISRC; Fein et al., 2001) and Beck Depression Inventory (BDI; Beck et al., 1996). Treatment consisted of three PE sessions provided on a weekly basis. Participants conducted weekly self-report measures and post-measures. All participants met an independent assessor who administered the Clinician-Administered PTSD Scale (CAPS-5; Weathers et al., 2013) to assess for PTSD 2 months after treatment completion.

### Measures

The Posttraumatic Stress Disorder Checklist (PCL-5; Blevins et al., 2015), a 20-item self-report measure, was used to assess PTSD symptoms after the treatment period. Other self-report measures that were used at post-treatment included the BDI (Beck et al., 1996) to assess for depressive symptoms, the World Health Organization Disability Assessment Schedule (WHODAS-12; Üstün et al., 2010) to asses general functioning, the Insomnia Severity Index (ISI; Bastien et al., 2001) to asses quality of sleep, and the Multidimensional Scale of Perceived Social Support (MSPSS; Zimet et al., 1990) to asses quality of social support. Diagnosis of PTSD and symptom severity was assessed using the CAPS-5 (Weathers et al., 2013) 2 months after treatment completion. We also assessed adverse events at each treatment session and at this 2-month follow-up.

In addition to the outcome measures above, we exploratory investigated if it is possible for rape victims to daily record the number of intrusive memories during treatment. This was done using a daily pen and paper diary where the participant was instructed to tick a box for the day and corresponding time period (morning/afternoon/evening/night) or indicate zero in the absence of intrusive memories. The diary was translated into Swedish by the first author and has been used in previous trials testing early provided psychological interventions (Horsch et al., 2017; Iyadurai et al., 2018).

### Intervention

Prolonged exposure is based on Foa and Kozak (1986) theory on emotional processing where PTSD symptoms are seen as pathological fear structures, activated by otherwise safe stimuli. This theory proposes that in order to reduce PTSD symptoms, the fear structure needs to be activated and corrective information made available. When patients start to avoid the memories or situations associated with the trauma, corrective information is not available. The aim with PE is therefore to break avoidance patterns and approach trauma-related stimuli, providing an opportunity for corrective information. It is hypothesized that if PE is provided in the early aftermath of trauma, it is possible to make swift modifications of the fear structure and manipulate the fear memory.

The PE treatment protocol was translated to Swedish by the first author in a previous trial after it was generously made available by the study authors of the Rothbaum et al. (2012) trial. The first author has been trained in PE by the treatment developer (Professor Edna Foa) and is a certified PE trainer. The therapists consisted of one psychotherapist and one clinical psychologist with extensive experience in treating rape victims and had access to supervision on demand.

The participants were provided with the first PE session within 72 h after the rape. During the first PE session, participants were provided with a rationale of the PE treatment and the function of avoidance behaviors as maintaining symptoms of post-traumatic stress. Subsequently imaginal exposure, where the patient was instructed to revisit the memory of the rape for 20–30 min (i.e., visualize the rape in their mind’s eye) and recounting the rape in present tense, was conducted together with the therapist. In order to address erroneous trauma-related cognitions, open-ended questions were used after the imaginal exposure. A voice recorder was used to record the imaginal exposure on which the patient was instructed to listen to the recording each day as homework. A technique to decrease arousal symptoms in the patient’s daily life, breathing retraining, was also taught to the patient. Subsequently, two additional 60-min sessions were provided to the participants. The aim of these two sessions was to review the homework assignments and conduct additional therapist-led imaginal exposure.

## Results

### Recruitment and Baseline Characteristics

A total of 191 patients underwent the pre-selection screening for eligibility of which 118 (61.7%) were immediately excluded due to the following reasons: >72 h had passed since the rape (36 patients; 18.8% of the screened sample), not fluent in Swedish (18 patients, 9.4% of the screened sample), <18 years of age (45 patients; 23.6% of the screened sample), and male gender (19 patients; 9.9% of the screened sample).

Seventy-three patients remained for the next phase of screening, using the digital medical records from the forensic examination. Nineteen patients (9.9% of the original sample) were not able to schedule an appointment with the therapist within the stipulated time frame for inclusion (common reasons were sickness, traveling, or not able to be reached by phone). Another nine patients (4.7% of the original sample) could not be scheduled within 72 h due to lack of available appointments in either the clinical psychologist or psychotherapists schedule. Ten additional patients (5.2% of the original sample) were excluded because they had no memory of the rape, and two patients (1%) were too physically ill and needed somatic care. Fourteen patients (7.3% of the original sample) had other serious psychiatric comorbidity and were thus excluded, and seven additional patients (3.7% of the original sample) did not want to be reached by the clinic personnel after the forensic examination. A total of 12 patients (6.3% of the original sample) were asked to participate in the study of which two patients declined further participation in the study.

Mean time from the traumatic event (rape) to the start of PE was 52.1 h (SD = 11.1). Baseline characteristics for the included participants are presented in Table 1.

**TABLE 1:** Baseline characteristics for included participants.

**Gender**	
Women, *n* (%)	10(100%)
**Age (years)**	
Age, year, mean (SD)	28 (6.8)
**Occupational status**	
Working, *n* (%)	7(70%)
Student, *n* (%)	1(10%)
On sick leave, *n* (%)	1(10%)
Actively seeking employment	1(10%)
**Traumatic event**	
Rape, one assailant, *n* (%)	8(80%)
Rape, multiple assailants, *n* (%)	2(20%)
Time from traumatic event to start of intervention, mean hours (SD)	52.1 (11.1)
**History of trauma and mental illness**	
Prior psychological trauma as an adult, *n* (%)	4(40%)
Prior psychological trauma as a child, *n* (%)	1(10%)
Prior psychological trauma both as a child and adult, *n* (%)	3(30%)
None	2(20%)
**Baseline measures**	
ISRC score, mean (SD)	39 (5.81)
BDI score, mean (SD)	29.3 (14.2)

*ISRC, Immediate Stress Reaction checklist; BDI, Beck Depression Inventory.*

### Treatment Acceptability and Data Attrition

Three of the included participants dropped out from the study after the second PE session. No reason for dropping out was given in two cases. The third participant stated being exposed to another traumatic event as a reason. Seven participants were provided with the full treatment protocol. Adherence to the treatment protocol was excellent, and all PE sessions were provided according to treatment protocol. Homework compliance was high in these seven participants, ranging from 85% completion between session 2 and 3 and 100% between session 1 and 2.

Data attrition was high at the 2-month follow-up; only six of the ten (60%) enrolled participants completed the CAPS-5 interview with the independent clinician. Data attrition was also high on the daily intrusion diary from session 1 to 3 where only three of ten (30%) participants had daily registrations the full treatment period.

### Outcome Data

The mean score on the PCL-5 was 49.9 points (SD = 9.5) at post-treatment (end of session 3) which had reduced to 38.8 points (SD = 14.5) at the 2-month follow-up. The corresponding number for the BDI was 21.6 points (SD = 7.1) at post-treatment and 18.3 points (SD = 9.9) at the 2-month follow-up.

The mean score on the CAPS-5 was 28.3 points (SD = 13.7) at the 2-month follow-up where three participants also received a diagnosis of PTSD and were offered standard PE treatment for PTSD. The mean ISI score was 12 points (SD = 4.48), the mean MSPSS point was 68 points (SD = 11.11), and the mean WHODAS score was 19.5 points (SD = 3.70) at the 2-month follow-up. No serious adverse events that could be attributed to the treatment were reported during the treatment or at the 2-month follow-up.

## Discussion

In this study, we hypothesized that a brief PE intervention provided in the early aftermath of rape would be a feasible and acceptable early psychological treatment for PTSD in regular Swedish care. Our hypotheses were partly supported: Of the 10 included individuals, seven participants completed the full PE protocol, and these individuals had high degree of treatment adherence. Additionally, results indicated clinically meaningful reductions in PTSD symptoms in these individuals. However, it is important to point out that the treated sample consisted of only a fraction (5.2%) of the total intake at the clinic during the study period.

As much as 40% of the screened sample was excluded due to the time criteria of 72 h. One well-reported factor in the literature is that only a small proportion of rape victims seek help from mental health professionals in the early aftermath of trauma (Campbell et al., 2001; Ullman, 2007; Zinzow et al., 2008) and there is a general delay from traumatic event and psychological treatment of 10 years (Kessler et al., 1995). One idea to increase scalability and to capture a larger population of rape victims would therefore be to extend the time criteria for early intervention and test if condensed PE is effective also after a longer time period since the traumatic event. Additionally, as much as 75% of the screened patients attended at the clinic after office hours and – as we did not have 24/7 coverage of clinical psychologists in this study – this may have led to unnecessary waiting times and exclusions for otherwise eligible participants. An interesting venue for future implementation research would be to investigate if it is possible to have psychologists around the clock also at emergency departments. This is to our knowledge very unusual in regular care and could be interesting to investigate further. At least nine (4.7%) eligible patients could not be scheduled within 72 h due to lack of available appointments in the clinical psychologist or licensed psychotherapists schedule and additional 19 (9.9%) patients were not able to schedule to early PE treatment due to other logistical reasons (e.g., traveling, not able to come to the clinic). Beside more psychologist coverage around the clock, one idea would be to develop complementary procedures to reach rape victims after office hours, e.g., posters in the waiting-room or automatized internet-screenings swiftly delivered via smart phone. Smart phone-based interventions have shown promise as preventive-, guidance-, and assessment tools for rape (Lindsay et al., 2013; Acosta et al., 2017; Narang et al., 2018), and a next step would be to also develop psychological interventions for victims of rape. Low-intensity treatments such as Internet-based cognitive behavior therapy have shown to be effective for the treatment of both PTSD and subthreshold symptoms and may be a feasible option for swift delivery of treatment for rape victims (Sijbrandij et al., 2016; Ennis et al., 2018).

Ten percent of the screened sample was not fluent in Swedish and therefore excluded. Translator assisted trauma focused CBT for PTSD has proven to be effective (d’Ardenne et al., 2007), and one future scientific direction would be to investigate if this could work also in an early intervention approach. A complementary way to solve linguistical barriers would be to develop more language free interventions. Recently, Iyadurai et al. (2018) showed, in a sample of motor vehicle accident victims, that a behavioral intervention including a memory reminder together with a visuospatial task (playing the computer game Tetris) could reduce the amount of intrusive memories the subsequent week after the trauma. Results also seem to extend to women traumatized by emergency cesarean section (Horsch et al., 2017) but it is still unclear if these findings can extend to rape victims.

About one-quarter of the screened sample was excluded due to the age criteria (at least 18 years old). Previous research indicate female adolescents as victims of sexual assault in a higher, disproportionately degree (Khadr et al., 2018). Adolescents are also overall more vulnerable to subsequent mental health problems after sexual assault than adults (Khadr et al., 2018), and studies has also indicated poorer educational outcomes for adolescent females exposed to sexual assault such as rape (Holmes and Sher, 2013; Martz et al., 2016). Although one recent trial showed that a PE package, delivered over 14 weeks, was effective in reducing full-blown PTSD symptoms for adolescents after sexual abuse (Foa et al., 2013), we are not aware of any research study investigating the efficacy of early psychological interventions for young rape victims. Consequently, developing early interventions for this particularly vulnerable group is imperative.

The Emergency Clinic for Rape Victims did only provide care to female rape victims during the main execution phase of this study which unfortunately excluded an additional 10% of the screened sample. About 4–10% of all reported rapes include a male victim (Siegel et al., 1987; Elliott et al., 2004; Snipes et al., 2017), and studies do indicate that male rape victims have higher degree of distress, psychiatric symptoms, and psychiatric hospitalizations than female rape victims (Kimerling et al., 2002; Tewksbury, 2007). Although one recent trial showed some promising effects of cognitive processing therapy for male rape victims (veterans) with PTSD (Mullen et al., 2014), other studies indicate that male rape victims are more treatment refractory (Galovski et al., 2013). To our knowledge, there has not been any attempts to deliver early psychological interventions for this particular patient group. Given that male rape victims may experience very high degree of stigma (Nasjleti, 1980; Rew and Esparza, 1990), it is important to develop low threshold scalable interventions for these individuals.

Twelve (6.2%) patients were finally asked to participate in the study, and two of these individuals declined participation. Of the 10 included participants, three participants dropped out of treatment. Homework assignment completion rate was high among the seven participants that underwent the full treatment, between 85 and 100%. One important challenge for future research is how to overcome the degree of data loss found in this study (40% of the included participants did not show up at the 2-month follow-up assessment). As previously discussed, digital innovations (e.g., smart phone apps, video-conference) may provide solutions to some of these problems. Several studies have shown promising results of smart phone assessments for several mental health problems (Arean et al., 2016) as well as for trauma-related symptoms (Price et al., 2017; van der Meer et al., 2017). One study did not find any statistical difference in assessing symptoms of PTSD using scores on the PCL-5 administered on smart phone or on pen and paper (Price et al., 2015). On the other hand, another study investigating the use of a smartphone diary in tracking autobiographical memories of personal events (Laughland and Kvavilashvili, 2018) showed disappointing results. Not only did the use of a smartphone diary not show any beneficial effects in recording memories, participants actually recorded less memories on the smart phone than on a paper and pen diary, concluding that further innovations may be needed (Laughland and Kvavilashvili, 2018).

The study comes with limitations. One major limitation is that the lack of a control group to control for spontaneous fluctuations. Although the treated participants on average had a clinical meaningful decline in PTSD symptoms, this symptom reduction may as well be explained by natural recovery. However, it is important to stress that the aim of this study was merely to assess feasibility and delivery of the PE protocol. Future studies should consequently use a parallel group design to investigate efficacy of this treatment with extended time criteria. Another limitation in this study was that the independent assessor was not blinded to the aim study hypothesis and time-point, and thus, the mean scores on the CAPS-5 should be interpreted with caution. We also used a “supervision on demand” approach which means that we did not make a systematic control for treatment fidelity of the intervention. Future research may want to investigate this issue in more detail and to investigate possible therapist drifts when providing early PE interventions. The low rate of eligible patients to recruit also poses a major limitation. Comparing these figures with previous studies on early interventions after trauma shows that a low rate of eligibility and recruitment is unfortunately common stressing the need for the field overall of finding ways around it (Rothbaum et al., 2012; Iyadurai et al., 2018; Maples-Keller et al., 2020).

To summarize, early provided PE seems to be an acceptable and deliverable intervention for rape victims. However, of the 191 screened patients, we were only able to include 10 participants (5.2% of the screened sample) in the study. The main reason for this was an inability to recruit participants in the stipulated time window for this study of 72 h. We suggest an alternative approach which includes offering the first intervention session when the patients present irrespective of time since rape and also to develop remotely delivered interventions. Complementary interventions, delivered through other formats (e.g., online), that are easily accessible – irrespective of gender, language barriers, and geographical distances – are clearly needed in order to swiftly reach the millions of rape victims around the globe.

## Data Availability Statement

The raw data supporting the conclusions of this article will be made available by the authors upon request given that the request comply with Swedish and EU laws regulating protection of identifiable data.

## Ethics Statement

The studies involving human participants were reviewed and approved by the Regional Ethical Review Board in Stockholm, Sweden (ID:2016/2194–31). The patients/participants provided their written informed consent to participate in this study.

## Author Contributions

KL and LN conducted the data collection. MB and EA performed the data analysis and interpretation, and first draft of the article. All authors contributed to the study design and writing of the article, and read and approved the final manuscript.

## Conflict of Interest

The authors declare that the research was conducted in the absence of any commercial or financial relationships that could be construed as a potential conflict of interest.

## References

[B1] Acierno R. Resnick H. S. Flood A. Holmes M. (2003). An acute post-rape intervention to prevent substance use and abuse. *Addict. Behav.* 28 1701–1715. 10.1016/j.addbeh.2003.08.043 14656554

[B2] Acosta F. Wister M. A. Hernandez-Nolasco J. A. Pancardo P. (2017). “Moving smartphones to send emergency messages during assaults,” in *Paper presented at the International Conference on Innovative Mobile and Internet Services in Ubiquitous Computing*, Cham: Springer.

[B3] Arean P. A. Hoa Ly K. Andersson G. (2016). Mobile technology for mental health assessment. *Dialogues Clin. Neurosci.* 18 163–169. 27489456 10.31887/DCNS.2016.18.2/parean PMC4969703

[B4] Bastien C. H. Vallieres A. Morin C. M. (2001). Validation of the Insomnia Severity Index as an outcome measure for insomnia research. *Sleep Med.* 2 297–307. 10.1016/s1389-9457(00)00065-4 11438246

[B5] Beck A. Steer R. Brown G. (1996). *Beck Depression Inventory: Manual (Swedish version).* Stockholm: Psykologiförlaget.

[B6] Blevins C. A. Weathers F. W. Davis M. T. Witte T. K. Domino J. L. (2015). The posttraumatic stress disorder checklist for dsm-5 (pcl-5): development and initial psychometric evaluation. *J. Trauma Stress* 28 489–498. 10.1002/jts.22059 26606250

[B7] Breslau N. Kessler R. C. Chilcoat H. D. Schultz L. R. Davis G. C. Andreski P. (1998). Trauma and posttraumatic stress disorder in the community: the 1996 detroit area survey of trauma. *Arch. Gen. Psychiatry* 55 626–632. 10.1001/archpsyc.55.7.626 9672053

[B8] Campbell R. Wasco S. M. Ahrens C. E. Sefl T. Barnes H. E. (2001). Preventing the “Second rape” rape survivors’ experiences with community service providers. *J. Interpers. Violence* 16 1239–1259. 10.1177/088626001016012002

[B9] Chen L. P. Murad M. H. Paras M. L. Colbenson K. M. Sattler A. L. Goranson E. N. (2010). Sexual abuse and lifetime diagnosis of psychiatric disorders: systematic review and meta-analysis. *Mayo Clin. Proc.* 85 618–629. 10.4065/mcp.2009.0583 20458101 PMC2894717

[B10] d’Ardenne P. Ruaro L. Cestari L. Fakhoury W. Priebe S. (2007). Does interpreter-mediated CBT with traumatized refugee people work? A comparison of patient outcomes in East London. *Behavi. Cogn. Psychother.* 35 293–301. 10.1017/s1352465807003645

[B11] Dartnall E. Jewkes R. (2013). Sexual violence against women: the scope of the problem. *Best Pract. Res. Clin. Obstet. Gynaecol.* 27 3–13. 10.1016/j.bpobgyn.2012.08.002 22940107

[B12] Elliott D. M. Mok D. S. Briere J. (2004). Adult sexual assault: prevalence, symptomatology, and sex differences in the general population. *J. Trauma Stress* 17 203–211. 10.1080/00131881.2019.1600376 15253092

[B13] Ennis N. Sijercic I. Monson C. M. (2018). Internet-delivered early interventions for individuals exposed to traumatic events: systematic review. *J. Med. Int. Res.* 20:e280. 10.2196/jmir.9795 30429113 PMC6300083

[B14] Fein J. A. Kassam-Adams N. Vu T. Datner E. M. (2001). Emergency department evaluation of acute stress disorder symptoms in violently injured youths. *Ann. Emerg. Med.* 38 391–396. 10.1067/mem.2001.118225 11574795

[B15] Foa E. B. Kozak M. J. (1986). Emotional processing of fear: exposure to corrective information. *Psychol. Bull.* 99 20–35. 10.1037/0033-2909.99.1.20 2871574

[B16] Foa E. B. McLean C. P. Capaldi S. Rosenfield D. (2013). Prolonged exposure vs supportive counseling for sexual abuse-related PTSD in adolescent girls: a randomized clinical trial. *JAMA* 310 2650–2657. 10.1001/jama.2013.282829 24368465

[B17] Galovski T. E. Blain L. M. Chappuis C. Fletcher T. (2013). Sex differences in recovery from PTSD in male and female interpersonal assault survivors. *Behav. Res. Ther.* 51 247–255. 10.1016/j.brat.2013.02.002 23510841 PMC3677761

[B18] Gisladottir A. Harlow B. L. Gudmundsdottir B. Bjarnadottir R. I. Jonsdottir E. Aspelund T. (2014). Risk factors and health during pregnancy among women previously exposed to sexual violence. *Acta Obstet. Gynecol. Scand.* 93 351–358. 10.1111/aogs.12331 24490826

[B19] Hannan S. M. Orcutt H. K. Miron L. R. Thompson K. L. (2017). Childhood sexual abuse and later alcohol-related problems: investigating the roles of revictimization, PTSD, and drinking motivations among college women. *J. Interpers. Violence* 32 2118–2138. 10.1177/0886260515591276 26130681

[B20] Holmes K. Sher L. (2013). Dating violence and suicidal behavior in adolescents. *Int. J. Adolesc. Med. Health* 25 257–261. 10.1515/ijamh-2013-0059 24006321

[B21] Horsch A. Vial Y. Favrod C. Harari M. M. Blackwell S. E. Watson P. (2017). Reducing intrusive traumatic memories after emergency caesarean section: a proof-of-principle randomized controlled study. *Behav. Res. Ther.* 94 36–47. 10.1016/j.brat.2017.03.018 28453969 PMC5466064

[B22] Iyadurai L. Blackwell S. E. Meiser-Stedman R. Watson P. C. Bonsall M. B. Geddes J. R. (2018). Preventing intrusive memories after trauma via a brief intervention involving Tetris computer game play in the emergency department: a proof-of-concept randomized controlled trial. *Mol. Psychiatry* 23 674–682. 10.1038/mp.2017.23 28348380 PMC5822451

[B23] Jina R. Thomas L. S. (2013). Health consequences of sexual violence against women. *Best Pract. Res. Clin. Obstet. Gynaecol.* 27 15–26. 10.1016/j.bpobgyn.2012.08.012 22975432

[B24] Kessler R. C. Chiu W. Demler O. Walters E. E. (2005). Prevalence, severity, and comorbidity of 12-month dsm-iv disorders in the national comorbidity survey replication. *Arch. Gen. Psychiatry* 62 617–627. 10.1001/archpsyc.62.6.617 15939839 PMC2847357

[B25] Kessler R. C. Sonnega A. Bromet E. Hughes M. Nelson C. B. (1995). Posttraumatic stress disorder in the national comorbidity survey. *Arch. Gen. Psychiatry* 52 1048–1060. 10.1001/archpsyc.1995.03950240066012 7492257

[B26] Khadr S. Clarke V. Wellings K. Villalta L. Goddard A. Welch J. (2018). Mental and sexual health outcomes following sexual assault in adolescents: a prospective cohort study. *Lancet Child Adolesc. Health* 2 654–665. 10.1016/s2352-4642(18)30202-5 30119759

[B27] Kimerling R. Rellini A. Kelly V. Judson P. L. Learman L. A. (2002). Gender differences in victim and crime characteristics of sexual assaults. *J. Interpers. Violence* 17 526–532. 10.1177/0886260502017005003

[B28] Laughland A. Kvavilashvili L. (2018). Should participants be left to their own devices? Comparing paper and smartphone diaries in psychological research. *J. Appl. Res. Mem. Cogn.* 7 552–563. 10.1016/j.jarmac.2018.09.002

[B29] Lindsay M. Messing J. T. Thaller J. Baldwin A. Clough A. Bloom T. (2013). Survivor feedback on a safety decision aid smartphone application for college-age women in abusive relationships. *J. Technol. Hum. Services* 31 368–388. 10.1080/15228835.2013.861784

[B30] Liu H. Petukhova M. V. Sampson N. A. Aguilar-Gaxiola S. Alonso J. Andrade L. H. (2017). Association of DSM-IV posttraumatic stress disorder with traumatic experience type and history in the World Health Organization World Mental Health Surveys. *JAMA Psychiatry* 74 270–281. 10.1001/jamapsychiatry.2016.3783 28055082 PMC5441566

[B31] Maples-Keller J. L. Post L. M. Price M. Goodnight J. M. Burton M. S. Yasinski C. W. (2020). Investigation of optimal dose of early intervention to prevent posttraumatic stress disorder: a multiarm randomized trial of one and three sessions of modified prolonged exposure. *Depress Anxiety* 37 429–437. 10.1002/da.23015 32248637 PMC7347250

[B32] Martz D. M. Jameson J. P. Page A. D. (2016). Psychological health and academic success in rural appalachian adolescents exposed to physical and sexual interpersonal violence. *Am. J. Orthopsychiatry* 86 594–601. 10.1037/ort0000174 27148751

[B33] McFarlane A. C. Atchison M. Rafalowicz E. Papay P. (1994). Physical symptoms in post-traumatic stress disorder. *J. Psychosom. Res.* 38 715–726. 7877126 10.1016/0022-3999(94)90024-8

[B34] Miller K. E. Cranston C. C. Davis J. L. Newman E. Resnick H. (2015). Psychological outcomes after a sexual assault video intervention: a randomized trial. *J. Forensic Nurs.* 11 129–136. 10.1097/jfn.0000000000000080 26291847

[B35] Mullen K. Holliday R. Morris E. Raja A. Suris A. (2014). Cognitive processing therapy for male veterans with military sexual trauma-related posttraumatic stress disorder. *J. Anxiety Disord.* 28 761–764. 10.1016/j.janxdis.2014.09.004 25260214

[B36] Narang J. Singhal C. Mathur A. Dubey A. K. Anil A. Pundir C. (2018). Naked-eye quantitative assay on paper device for date rape drug sensing via smart phone APP. *Vacuum* 153 300–305. 10.1016/j.vacuum.2018.03.056

[B37] Nasjleti M. (1980). Suffering in silence: the male incest victim. *Child Welfare* 59 269–275. 7371436

[B38] NCK (2014). *En Befolkningsundersökning om Kvinnors och Mäns Våldsutsatthet Samt Kopplingen till Hälsa Rapport Rapport.* Uppsala: Uppsala Unviersity.

[B39] Price M. Kuhn E. Hoffman J. E. Ruzek J. Acierno R. (2015). Comparison of the PTSD Checklist (PCL) administered via a mobile device relative to a paper form. *J. Trauma Stress* 28 480–483. 10.1002/jts.22037 26375277

[B40] Price M. van Stolk-Cooke K. Ward H. L. O’Keefe M. Gratton J. Skalka C. (2017). Tracking post-trauma psychopathology using mobile applications: a usability study. *J. Technol. Behav. Sci.* 2 41–48. 10.1007/s41347-016-0008-9 29109968 PMC5669390

[B41] Resnick H. Acierno R. Holmes M. Kilpatrick D. G. Jager N. (1999). Prevention of post-rape psychopathology: preliminary findings of a controlled acute rape treatment study. *J. Anxiety Disord.* 13 359–370. 10.1016/s0887-6185(99)00010-9 10504107

[B42] Resnick H. Acierno R. Waldrop A. E. King L. King D. Danielson C. (2007). Randomized controlled evaluation of an early intervention to prevent post-rape psychopathology. *Behav. Res. Ther.* 45 2432–2447. 10.1016/j.brat.2007.05.002 17585872 PMC2040305

[B43] Resnick H. S. Acierno R. Amstadter A. B. Self-Brown S. Kilpatrick D. G. (2007). An acute post-sexual assault intervention to prevent drug abuse: updated findings. *Addict. Behav.* 32 2032–2045. 10.1016/j.addbeh.2007.01.001 17275198 PMC1986828

[B44] Rew L. Esparza D. (1990). Barriers to disclosure among sexually abused male children. Implications for nursing practice. *J. Child Adolesc. Psychiatr. Ment. Health Nurs.* 3 120–127. 10.1111/j.1744-6171.1990.tb00458.x 2213519

[B45] Rothbaum B. O. Kearns M. C. Price M. Malcoun E. Davis M. Ressler K. J. (2012). Early intervention may prevent the development of posttraumatic stress disorder: a randomized pilot civilian study with modified prolonged exposure. *Biol. Psychiatry* 72 957–963. 10.1016/j.biopsych.2012.06.002 22766415 PMC3467345

[B46] Scott K. M. Koenen K. C. King A. Petukhova M. V. Alonso J. Bromet E. J. (2018). Post-traumatic stress disorder associated with sexual assault among women in the WHO World Mental Health Surveys. *Psychol. Med.* 48 155–167. 10.1017/s0033291717001593 28625214 PMC5896282

[B47] Siegel J. M. Sorenson S. B. Golding J. M. Burnam M. A. Stein J. A. (1987). The prevalence of childhood sexual assault. The Los Angeles Epidemiologic Catchment Area Project. *Am. J. Epidemiol.* 126 1141–1153. 3500638 10.1093/oxfordjournals.aje.a114752

[B48] Sijbrandij M. Kunovski I. Cuijpers P. (2016). Effectiveness of Internet-delivered cognitive behavioral therapy for posttraumatic stress disorder: a systematic review and meta-analysis. *Depress. Anxiety* 33 783–791. 10.1002/da.22533 27322710

[B49] Snipes D. J. Calton J. M. Green B. A. Perrin P. B. Benotsch E. G. (2017). Rape and posttraumatic stress disorder (PTSD): examining the mediating role of explicit sex-power beliefs for men versus women. *J. Interpers. Violence* 32 2453–2470. 10.1177/0886260515592618 26141347

[B50] Stenson K. Heimer G. Lundh C. Nordstrom M. L. Saarinen H. Wenker A. (2003). Lifetime prevalence of sexual abuse in a Swedish pregnant population. *Acta Obstet. Gynecol. Scand.* 82 529–536. 10.1034/j.1600-0412.2003.00111.x 12780423

[B51] Tewksbury R. (2007). Effects of sexual assaults on men: physical, mental and sexual consequences. *Int. J. Men’s Health* 6 22–35. 10.3149/jmh.0601.22

[B52] Tiihonen M. A. Backstrom T. Sondergaard H. P. Helstrom L. (2014). Identifying risk factors for PTSD in women seeking medical help after rape. *PLoS One* 9:e111136. 10.1371/journal.pone.0111136 25340763 PMC4207776

[B53] Ullman S. E. (2007). Mental health services seeking in sexual assault victims. *Women & Therapy* 30 61–84. 10.1300/j015v30n01_04

[B54] Üstün T. B. Kostanjsek N. Chatterji S. Rehm J. (2010). *Measuring Health and Disability: Manual for WHO Disability Assessment Schedule WHODAS 2.0.* Geneva: World Health Organization.

[B55] van der Meer C. A. I. Bakker A. Schrieken B. A. L. Hoofwijk M. C. Olff M. (2017). Screening for trauma-related symptoms via a smartphone app: the validity of smart assessment on your mobile in referred police officers. *Int. J. Methods Psychiatr. Res* 26:e1579. 10.1002/mpr.1579 28948699 PMC5639363

[B56] Walsh K. Gilmore A. K. Frazier P. Ledray L. Acierno R. Ruggiero K. J. (2017). A randomized clinical trial examining the effect of video-based prevention of alcohol and marijuana use among recent sexual assault victims. *Alcohol. Clin. Exp. Res.* 41 2163–2172. 10.1111/acer.13505 28940320 PMC5711597

[B57] Weathers F. Blake D. Schnurr P. Kaloupek D. Marx B. Keane T. (2013). *The clinician-administered PTSD scale for DSM-5 (CAPS-5).* Available online at www.ptsd.va.gov (accessed April 7, 2020). 10.1037/pas0000486 PMC5805662 28493729

[B58] Wolitzky-Taylor K. B. Resnick H. S. McCauley J. L. Amstadter A. B. Kilpatrick D. G. Ruggiero K. J. (2011). Is reporting of rape on the rise? A comparison of women with reported versus unreported rape experiences in the National Women’s Study-Replication. *J. Interpers. Violence* 26 807–832. 10.1177/0886260510365869 20522886

[B59] Zimet G. D. Powell S. S. Farley G. K. Werkman S. Berkoff K. A. (1990). Psychometric characteristics of the multidimensional scale of perceived social Support. *J. Pers. Assess.* 55 610–617. 10.1080/00223891.1990.9674095 2280326

[B60] Zinzow H. M. Grubaugh A. L. Frueh B. C. Magruder K. M. (2008). Sexual assault, mental health, and service use among male and female veterans seen in Veterans Affairs primary care clinics: a multi-site study. *Psychiatry Res.* 159 226–236. 10.1016/j.psychres.2007.04.008 18423615

